# Stipules in Apocynaceae: an ontogenetic perspective

**DOI:** 10.1093/aobpla/plw083

**Published:** 2017-02-10

**Authors:** Natalie do Valle Capelli, Bruna Alonso Rodrigues, Diego Demarco

**Affiliations:** 1 Departamento de Botânica, Instituto de Biociências, Universidade de São Paulo, São Paulo 05508-090, Brazil

**Keywords:** Apocynaceae, colleters, evolution, leaf structure, ontogeny, Rubiaceae, stipules

## Abstract

Stipules are leaf structures common in many groups of plants that can take a variety of forms. In Gentianales, interpetiolar stipules are considered a synapomorphy of Rubiaceae; however, some reports in the literature refer to their presence in other families. The goal of this study was to analyze the development of leaf primordia to investigate the possible presence of reduced or modified stipules in Apocynaceae. Shoot apices of 12 genera were analyzed under light and scanning electron microscopy comparatively with one species of Rubiaceae. Early in their development, leaf primordia form two lateral expansions at the base of the petiole (stipules) that give rise to colleters in 11 of the 12 genera of Apocynaceae studied, similarly to the Rubiaceae species. The basal genera have pairs of stipules modified into colleters positioned laterally to the petiole, while other species belonging to the derived subfamilies have interpetiolar stipules that each project towards the opposite stipule and merge, forming a sheathing stipule and from this arc the interpetiolar colleters originate. The ontogenetic study proved for the first time that Apocynaceae is a stipulate family whose stipules are modified into colleters and their absence might be a secondary loss, changing the interpretation of stipule evolution in Gentianales.

## Teaser

Stipules are leaf structures common in many groups of plants that can take a variety of forms. In Gentianales, interpetiolar stipules are considered a synapomorphy of Rubiaceae but in this study we detected two lateral expansions at the base of the leaf primordia (stipules) that give rise to colleters in many genera of Apocynaceae, similarly to Rubiaceae species. Through ontogenetic study it was possible to prove for the first time that Apocynaceae is a stipulate family whose stipules are modified into colleters, and their absence might be a secondary loss, changing the interpretation of stipule evolution in Gentianales.

## Introduction

Stipules are usually small, inconspicuous projections of the petiole base of leaves with protective function mainly in the bud, and there might be other functions as well. Often they appear as a pair of leafy appendages located on each side of the leaf base, but in some plants they may be modified into thorns or glands (Sinnott and Bailey 1914; Simpson 2006).

Stipules occur in many families of Eudicots and are especially important in some orders, such as Gentianales, which is composed of the families Apocynaceae, Gelsemiaceae, Gentianaceae, Loganiaceae and Rubiaceae (APG III 2009). Among these families, the presence of interpetiolar stipules is a typical characteristic of Rubiaceae, considered a synapomorphy of this family and a striking feature used to recognize the family (Robbrecht 1988; Bremer and Struwe 1992; Weberling 2006). However, there are some records in the literature of stipules in the other families, as in Apocynaceae cited by Mitra (1950), in Loganiaceae by Bremer and Struwe (1992) and Keller (1996), in Gelsemiaceae by Stevens (2001), and in Gentianaceae by Grant and Weaver (2003). Furthermore, Bremer and Struwe (1992) considered the interpetiolar stipules as an ancestral character for Gentianales families (symplesiomorphy), and the absence of such structure is probably due to reversion.

Those reports raise doubts about the occurrence and development of stipules in this group, especially in relation to Apocynaceae, generally characterized as not having stipules (Endress and Bruyns 2000). Apocynaceae comprise around 366 genera grouped within five subfamilies, 25 tribes and 49 subtribes (Endress *et al.* 2014) and around 5000 species (Endress 2004; Endress *et al.* 2007). The species may be vines, trees, shrubs or herbs, all having in common the presence of latex in their organs. The leaves are simple, opposite (or eventually alternate), sometimes reduced without stipules (Thomas and Dave 1991; Marcondes-Ferreira 1999; Judd *et al.* 2002; Simpson 2006).

In spite of the centuries of knowledge of the group and several structural studies performed in Apocynaceae, few studies have suggested the presence of morphologically distinguishable stipules in Apocynacean species (Sinnott and Bailey 1914; Mitra 1950; Meve and Albers 1990; Bruyns 2000), with Endress and Bruyns (2000) describing the rare occurrence of small, deciduous stipules. Several authors have also noted a possible stipular origin of some interpetiolar colleters (Woodson and Moore 1938; Bruyns 2000; Rio *et al.* 2002; Martins *et al.* 2010; Martins 2012; Canaveze and Machado 2015), but these records referred to this possible stipular origin based on colleter position and did not analyze the whole development of the leaf and the gland. Actually, Mitra (1950) is the only researcher to perform an ontogenetic study in one species of Apocynaceae, but the species studied is well-known for having morphologically distinguishable stipules in intrapetiolar position.

Colleters are secretory structures present in vegetative and/or reproductive organs that produce a sticky fluid, composed only of mucilage and/or lipophilic compounds, which protects meristems (Fahn 1979, 1990; Thomas 1991). In Apocynaceae, the colleter generally is a non-vascularized emergence occurring on the leaf, cotyledons, bracts, bracteoles, sepals, and corolla (Thomas 1991; Sennblad *et al.* 1998; Canaveze and Machado 2015) whose secretion may protect buds and developing organs against desiccation, herbivores and/or fungal proliferation (Demarco 2008).

On the leaves, colleters were recorded on the apex or base of the petiole and along the margin and/or midrib of the lamina in many genera of Apocynaceae belonging to all five subfamilies (Woodson and Moore 1938; Thomas 1991; Thomas and Dave 1991; Sennblad *et al.* 1998; Endress and Bruyns 2000). However, some of these colleters could indeed have stipular origin, evidenced by their similar position on the lateral portion of the base of the leaf petiole (Demarco 2008). This possibility is reinforced by the observation of colleters originating from the adaxial face of stipules in Rubiaceae species (Majumdar and Pal 1958; Robbrecht 1988; Klein *et al.* 2004; Vitarelli and Santos 2009; Miguel *et al.* 2009).

The homology among the traits of different taxa may be identified by different survey techniques, but when the morphology is profoundly altered, the only studies on the developmental mechanisms are able to perceive their common origin (Wake *et al.* 2011). This is because understanding the morphological evolution is necessary to analyze the ontogenetic processes, since evolutionary changes in the developmental patterns of related taxa result in morphological diversification (Doust and Kellogg 2002; Specht and Howarth 2015). Therefore, anatomical and morphological studies have been very useful to identify homologies in both vegetative and floral organs (Aguiar-Dias *et al.* 2011; Cruz *et al.* 2015; Gama *et al.* 2016).

The goal of this study was to verify the presence of reduced or modified stipules in the leaves of 12 genera of Apocynaceae belonging to different subfamilies, focusing on the beginning of leaf development and the ontogeny of interpetiolar colleters occurring on the petiole base to evaluate their possible stipular nature. One species of Rubiaceae was selected to compare the colleter/stipule origin.

## Methods

Twelve species belonging to 12 genera were selected for this study (Table 1) to represent both early- and late-diverging clades of Apocynaceae. Vouchers were deposited in the herbarium of the Universidade Estadual de Campinas (UEC). Also, one species of Rubiaceae, *Mussaenda erythrophylla*, was sampled at Universidade de São Paulo, São Paulo, SP, Brazil for comparison.

**Table 1.** Species selected for this study and their collection sites.

Species	Subfamily	Collection site (Brazil)	Collector number (Herbarium)
*Allamanda schottii* Pohl	Rauvolfioideae	Universidade de São Paulo, São Paulo (SP)	N. V. Capelli 1 (SPF)
*Aspidosperma australe* Müll. Arg.		Universidade Estadual de Campinas, Campinas (SP)	D. Demarco 10 (UEC)
*Thevetia peruviana* K.Schum.		Universidade de São Paulo, São Paulo (SP)	N. V. Capelli 2 (SPF)
*Mandevilla tenuifolia* (J.C.Mikan) Woodson	Apocynoideae	Parque Nacional da Serra do Cipó, Santana do Riacho (MG)	N. V. Capelli 3 (SPF)
*Secondatia densiflora* A.DC.		Parque Nacional da Serra do Cipó, Santana do Riacho (MG)	N. V. Capelli 4 (SPF)
*Asclepias curassavica* L.	Asclepiadoideae	Parque Estadual da Serra do Mar - Núcleo Picinguaba, Ubatuba (SP)	D. Demarco 52, 66, 68 (UEC)
*Blepharodon bicuspidatum* E. Fourn.		Reserva Biológica e Estação Experimental de Mogi-Guaçu, Mogi-Guaçu (SP)	D. Demarco 7, 11, 14 (UEC)
*Ditassa gracilis* Hand.-Mazz.		Parque Nacional da Serra do Cipó, Santana do Riacho (MG)	N. V. Capelli5 (SPF)
*Fischeria stellata* E. Fourn.		Parque Estadual da Serra do Mar - Núcleo Picinguaba, Ubatuba (SP)	D. Demarco 58, 60 (UEC)
*Matelea denticulata* (Vahl) Fontella and E.A. Schwarz.		Parque Estadual da Serra do Mar - Núcleo Picinguaba, Ubatuba (SP)	D. Demarco 37, 38 (UEC)
*Oxypetalum banksii* subsp. *banksii* Roem. and Schult.		Parque Estadual da Serra do Mar - Núcleo Picinguaba, Ubatuba (SP)	D. Demarco 57, 70 (UEC)
*Peplonia axillaris* (Vell.) Fontella & Rapini		Parque Estadual da Serra do Mar - Núcleo Picinguaba, Ubatuba (SP)	D. Demarco 35, 48, 49 (UEC)

Shoot apices were collected and promptly fixed in FAA (formalin, acetic acid, alcohol) for 24h (Johansen 1940) or BNF (buffered neutral formalin) in sodium phosphate buffer 0.1M pH 7.0 (Lillie 1965) and subsequently stored in ethanol 70  %. The isolated materials were dehydrated in a butyl series, embedded in Paraplast (Fisher Healthcare, Houston, Texas, USA) and transversely and longitudinally sectioned with 10 µm thick in a Microm HM340E rotary microtome (Microm International, Walldorf, Germany). The sections were stained with astra blue and safranin (Gerlach 1984), and the blades were mounted in resin Permount (Fisher Scientific, Pittsburgh, Pennsylvania, USA).

The anatomical analyses were performed under a Leica DMBL light microscope (Leica Microsystems, Wetzlar, Germany) using the Scan System Images (IM50).

## Results

### Apocynaceae

**Figure 1:**
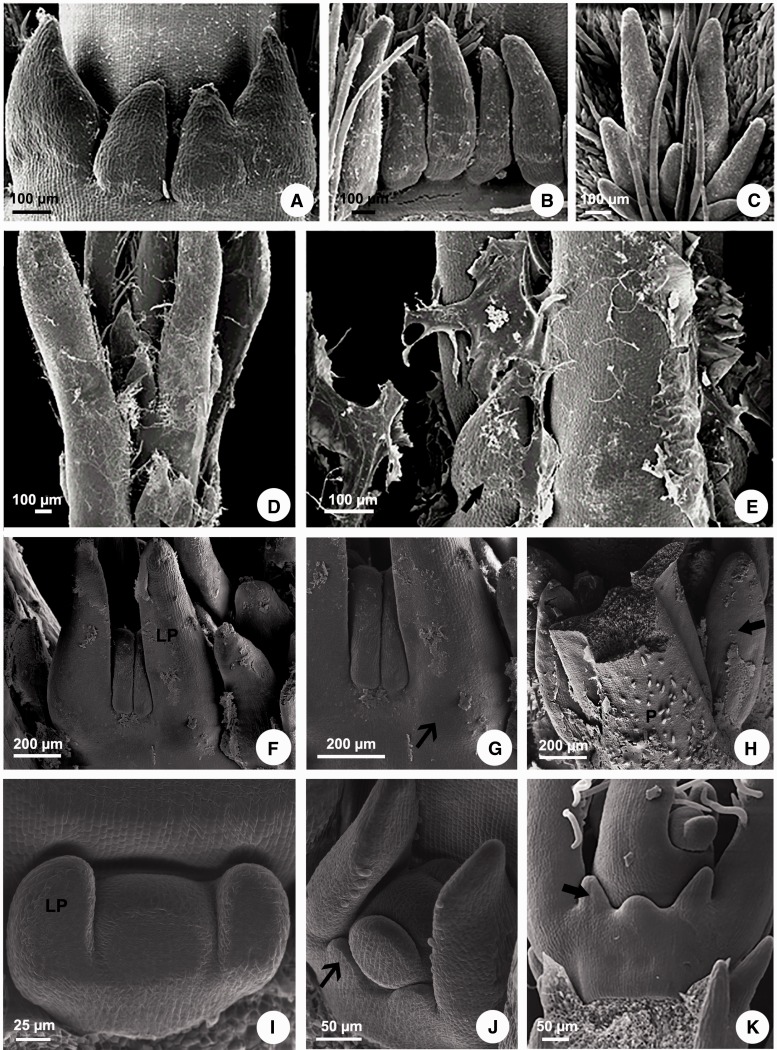
Scanning electron microscopy of colleters in Apocynaceae. (A, D, E) *Peplonia axillaris*. (B) *Asclepias curassavica*. (C) *Fischeria stellata*. (F–H) *Allamanda schottii*. (I–K) *Blepharadon bicuspidatum*. (A) Stipular colleters. (B) Petiolar colleters. (C) Laminar colleters. (D) Colleters in the shoot apex. (E) Colleters secretion in the shoot apex. (F–K) Initiation of stipules from leaf primordia. (F, G, J) Projection of the stipules from leaf primordia base. (H) Colleters originated from stipules laterally to the petiole. (I) Leaf primordia without stipules. (K) Colleters origin from sheathing stipules. LP = leaf primordium. P = petiole. Narrow arrow = stipule. Large arrow = stipular colleter.

**Figure 2:**
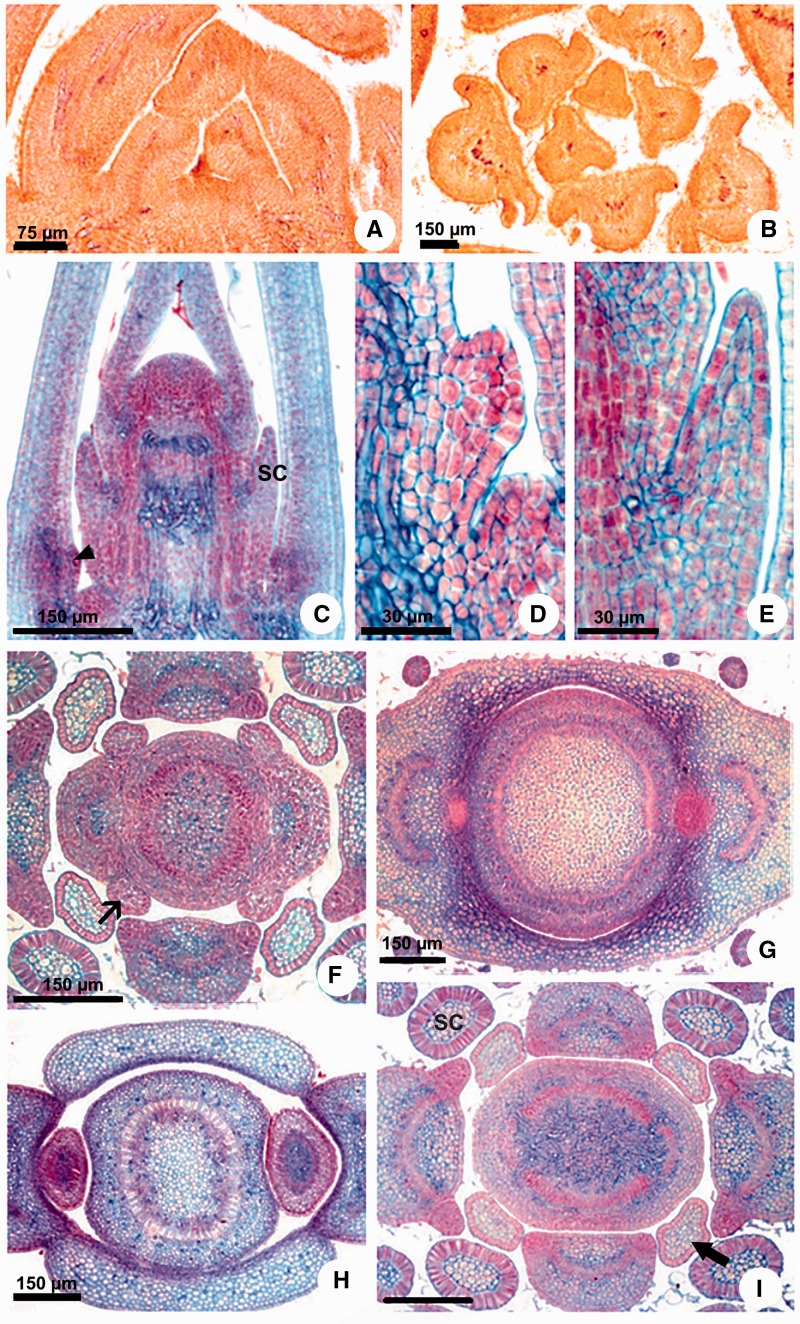
Stipule and colleter development in Apocynaceae. (A, B) *Aspidosperma australe*. (C–E, H) *Peplonia axillaris*. (F–G, I) *Asclepias curassavica.* (A, C–E) Longitudinal sections. (B, F–I) Transversal sections. (A, B) Leaf primordia without stipules and colleters. (C) Shoot apex with developing colleters. (D) Detail of the petiolar colleter initiation. (E) Detail of the stipular colleter in pre-secretory phase. (F) Projection of the stipules from leaf primordia base. (G, H) Sheathing stipules. (I) Stipular colleters in secretory phase and in pre-secretory phase. SC = stipular colleter. Narrow arrow = stipule. Large arrow = colleter in pre-secretory phase. Arrowhead = petiolar colleter initiation.

***Morphology*:** The leaves of all species have opposite phyllotaxis, being alternate only in *Aspidosperma*. The morphological analysis of leaves was not able to detect any stipule in the species of Apocynaceae studied, but colleters are easily observed on leaves of all species (Fig. 1A–E), except in *Aspidosperma*, whose leaves do not have any kind of external gland (Fig. 2A and B).

Colleters were observed at various locations (Table 2). Depending on the species, they occur at the lateral base of the petiole (stipular colleters; Fig. 1A), on the distal or the proximal position of the petiole (petiolar colleters; Fig. 1B) and/or on leaf blade (laminar colleters; Fig. 1C).

**Table 2.** Colleter position and morphology in Apocynaceae. + = presence; − = absence; s = stipular colleter; p = petiolar colleter; l = laminar colleter.

Species	Subfamily	Colleter position	Colleter peduncle
*Allamanda schottii*	Rauvolfioideae	stipule, petiole	+
*Aspidosperma australe*		−	−
*Thevetia peruviana*		stipule, petiole	−
*Mandevilla tenuifolia*	Apocynoideae	stipule, petiole	+ (s) − (p)
*Secondatia densiflora*		stipule, petiole	+
*Asclepias curassavica*	Asclepiadoideae	stipule, petiole	+
*Blepharodon bicuspidatum*		stipule, petiole	+
*Ditassa gracilis*		stipule, lamina	+ (s) − (l)
*Fischeria stellata*		stipule, petiole, lamina	+ (s, p) − (l)
*Matelea denticulata*		stipule, lamina	+ (s) − (l)
*Oxypetalum banksii*		stipule, lamina	+
*Peplonia axillaris*		stipule, petiole	+ (s) − (p)

**Figure 3:**
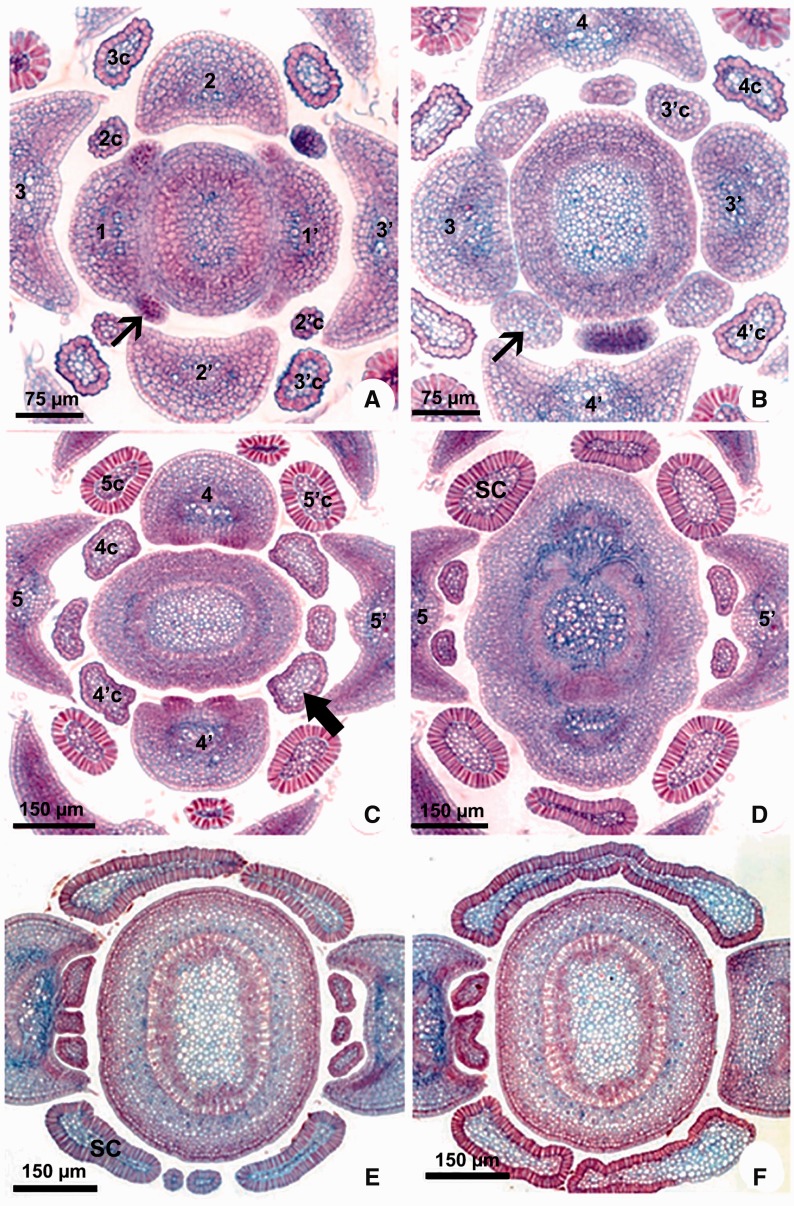
Stipule development in Apocynaceae. Transversal sections. (A–D), *Blepharodon bicuspidatum*. (E, F) *Peplonia axillaris.* (A–D) Serial sections of shoot apex showing stipule development giving rise to colleters. (E, F) Serial sections of the sheathing stipules modified into colleter. The numbers correspond to the ontogenetic sequence of the leaves formation (1–5) and their respective colleters (c). SC = stipular colleter. Narrow arrow = stipule. Large arrow = colleter in pre-secretory phase.

**Figure 4:**
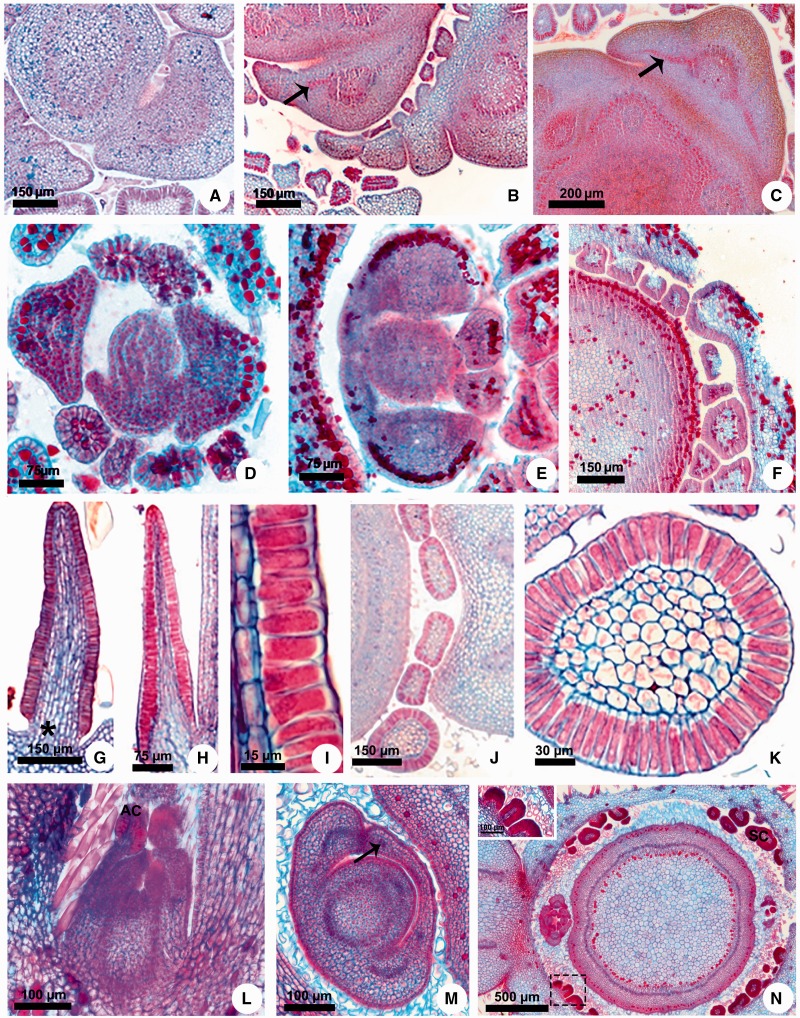
Stipule development and colleter structure in Apocynaceae and Rubiaceae. (A) *Secontatia densiflora.* (B, C) *Thevetia peruviana*. (D) *Mandevilla tenuifolia*. (E, F) *Secondatia densiflora*. (G) *Fisheria stellata*. (H, I) *Peplonia axillaris*. (J, K) *Asclepias curassavica*. (L–N) *Mussaenda erythrophylla*. (A–F, J, K, M, N) Transversal sections. (G–I, L) Longitudinal sections. (A–C) Projection of the vascularized stipules as a pair laterally to the petiole. (D) Projection of the stipule from leaf primordium base. (E) Fusion of the sheathing stipules of a node. (F, N) Sheathing stipules giving rise to colleters. (G, H) General view of colleters. (I) Detail of the secretory epidermis. (J) Stipular and petiolar colleters. (K) Detail of the stipular colleter. (L) General view of the shoot apex. (M) Sheathing stipules with vascular bundles. (N) Detail of the stipular colleters (inset). * = colleter peduncle. Arrow = vascular bundle of the stipule. AC = apical colleter of the stipule. SC = stipular colleter.

***Ontogeny*:** The ontogenetic analyses revealed that the laminar and petiolar colleters are simply enations of restricted points of the leaf (Fig. 2C), but all interpetiolar colleters are derived from two groups of meristematic cells which develop from each side of the petiole base (stipules) early in the development of the leaf primordia (Figs. 1F–K, 2D–I, 3A–F, 4A–F).

The meristematic stipules are present in all genera sampled with the exception of *Aspidosperma*, but in *Allamanda* and *Thevetia* (basal genera) the stipules grow as a pair laterally to the petiole and become glandular with the base fused to the stem (Fig. 4A–C). In all other genera, the stipules of the two leaves of a node grow toward each other and merge, forming a stipular sheathing from which the interpetiolar colleters originate (Figs. 2G and H, 3E and F, 4E and F). After the fusion of the meristematic stipules forms an arc, it fuses with the stem (Fig. 3D).

As the stipules emerge early in leaf development, the interpetiolar colleters are always the first to differentiate on the leaf primordia (Fig. 1F and G, I–K) and can already be seen in the pre-secretory phase in the primordia of the second or third node (Fig. 2E). They occur close to the petiole in *Allamanda*, *Asclepias*, *Ditassa*, *Fischeria*, *Mandevilla*, *Matelea*, *Oxypetalum*, and *Thevetia* (Fig. 2I) or continuously on the sheathing stipules between the petioles of the two leaves of a node in *Blepharodon, Peplonia* and *Secondatia* (Figs 1A, 3C–F).

***Anatomy*:** These colleters are glandular emergences finger-shaped or of the standard type (Fig. 4G–I), having a non-secreting peduncle limited to a few cell layers (Fig. 4G, asterisk) and an elongated secretory head, consisting of an axis of parenchyma covered by a uniseriate secretory palisade epidermis (Fig. 4G–K).

Vascular tissues are absent in all colleters analyzed (Fig. 4K). However, the presence of vascularization was variable in the base of the stipules near the petiole. *Allamanda* and *Thevetia*, whose stipules are short and lateral to the petiole, have one short vascular bundle derived from the border of the petiole vascular system (Fig. 4B and C, arrow). In the other genera, no vestige of vascular tissues was observed in the sheathing stipules nor any divergence of vascular bundles from the petiole in the direction towards the stipules (Figs 2G and H, 3F, 4E and F).

### Rubiaceae

***Morphology*:** *Mussaenda* also have decussate leaves with morphologically distinct interpetiolar stipules and colleters. The colleters occur on the adaxial face of the stipules (stipular colleters) near the petiole, and the tip of the stipules is also modified in colleter (Fig. 4L–N).

***Ontogeny*:** The interpetiolar stipules of *Mussaenda* are formed in exactly in the same way as in Apocynaceae: the meristematic stipules emerge laterally from the base of the two leaf primordia of a node, growing towards each other and merging in a stipular sheathing with proximal vascular bundles derived from the petiole vasculature (Fig. 4M). After this fusion and the initial development of the leafy stipules, the colleters originate.

***Anatomy*:** Colleters of *Mussaenda* on the interpetiolar stipule base are of the standard type, similar to colleters of Apocynaceae, having a short, non-secreting peduncle and a secretory head composed of a parenchyma core covered by a uniseriate secretory palisade epidermis (Fig. 4N inset). The colleter of the stipule tip has the same histology as the others (Fig. 4L).

All interpetiolar colleters are non-vascularized, including the colleter of the stipule tip. The stipules of *Mussaenda* have a leafy structure, composed of a uniseriate epidermis, chlorophyll parenchyma along the whole extension of the stipular arc, and few vascular bundles near the petiole at the stipules base (Fig. 4M) which ramify in several vascular branches toward the apex. Despite the proximity of the stipule vasculature in relation to the interpetiolar colleters, the vascular tissues do not enter the secretory portion (Fig. 4N).

## Discussion

**Figure 5:**
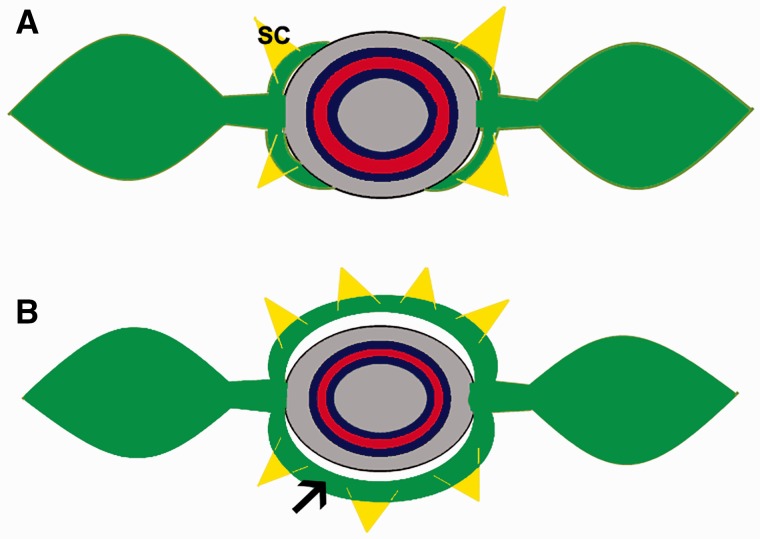
Drawing of nodal morphology in Apocynaceae. (A) Drawing of a node with short stipules based on *Allamanda* and *Thevetia*. (B) Drawing of a node with sheathing stipules. Arrow = stipule. SC = stipular colleter.

Our study proved that the family Apocynaceae is stipulated, but their stipules can be identified only through ontogenetic study. Apparently, the absence of stipules might be a secondary loss since these modified leaf structures were identified in 11 genera belonging to three subfamilies of Apocynaceae. Based on the phylogeny, the basal genera have stipules laterally to the petiole and most genera have a stipular sheathing formed by merged stipules forming an arc linking the petioles (Fig. 5), which gives rise to interpetiolar colleters in a similar way to that of Rubiaceae. The two types of stipule insertion are also found in Rubiaceae. Although the stipules are mostly fused forming an interpetiolar structure on either side of the stem, there are a few genera with the supposed plesiomorphic condition, *i*.*e*. each petiole with two stipules, one at each side (Robbrecht 1988).

The presence of colleters was already recognized in the order Gentianales, and their structure has been extensively described (Robbrecht 1988; Thomas 1991; Appezzato-da-Gloria and Estelita 2000; Rio *et al.* 2002; Demarco 2005, 2008; Vitarelli and Santos 2009; Martins *et al.* 2010; Martins 2012; Canaveze and Machado 2015, among others). This gland has taxonomic and phylogenetic importance to the order (Bremer and Struwe 1992), but its origin was still poorly studied in Apocynaceae (Mitra 1950).

Although some researchers have suggested that colleters could be considered as stipular structures (Woodson 1936; Woodson and Moore 1938; Rio *et al.* 2002; Martins *et al.* 2010; Martins 2012; Canaveze and Machado 2015), these studies are based mainly on structural analysis of adult organs. Few studies have addressed some aspects of their development (Canaveze and Machado 2015).

Furthermore, the stipular origin has been opposed by Thomas (1991) and Thomas and Dave (1991) because colleters occur in the five subfamilies of Apocynaceae with a predominant incidence of leaf colleters in petiolar or laminar position and/or in reproductive organs, such as the calyx and corolla (Thomas 1991), where stipules could not occur. However, calycine colleters in Apocynaceae do not arise from the base of the sepals, but from the adaxial face of a higher portion of the connate calyx (Demarco 2008), resembling the origin of laminar colleters in leaves. Furthermore, the same type of gland may have more than one origin (Fahn 1979), such as the colleters in Rubiaceae (Robbrecht 1988).

In addition, the colleters may have different morphologies. The standard type has a short peduncle and a secretory head composed of a parenchyma axis surrounded by an epidermis of elongated secretory cells. This is the most common type found in Apocynaceae, but it is not the only one. Many morphological types have recently been described (Demarco 2005, 2008; Martins *et al.* 2010; Martins 2012; Canaveze and Machado 2015), and they vary in number, including the species studied in the present work (Thomas 1991) and in relation to the presence or absence of peduncle when considering different species or colleters in different positions in the same organ or the same individual.

Most genera of Rubiaceae described by Robbrecht (1988), Thomas (1991), Weberling (2006), Vitarelli and Santos (2009), Miguel *et al.* (2009) and Coelho *et al.* (2013) have a stipular position of the colleters. The major difficulty of acknowledging the stipular origin of the colleters in Apocynaceae was probably due to the great diversity of positions. Although many colleters can be found around the base of the leaf petiole, few of them have a stipular origin. However, some previous studies have described stipules in the families of Gentianales. Meve and Albers (1990) and Bruyns (2000) recorded possible stipular rudiments in Stapelieae (Asclepiadoideae). Keller (1996) describes stipules as expansions of the petiole base forming a stipular sheath in Loganiaceae, and Grant and Weaver (2003) observed free interpetiolar and deciduous stipules in Gentianaceae. In addition, Bremer and Struwe (1992) claimed that Gentianales is a monophyletic group based on several unique morphological and chemical characters, and considered the interpetiolar stipules as an ancestral trait of the order which was lost (reversion), apparently in most Apocynaceae (Endress and Bruyns 2000) and Gentianaceae (Grant and Weaver 2003).

Another difficulty is the absence of a vascular system in these stipules modified into interpetiolar colleters of Apocynaceae, a feature not shared with stipules of other families, including Rubiaceae (Vitarelli and Santos 2009). Colleters in Apocynaceae generally do not have vascular tissue (Woodson and Moore 1938), but vascularized colleters have been described on leaves of *Mandevilla*, *Odontadenia, Prestonia* and *Temnadenia* (Appezzato-da-Glória and Estelita 2000; Rio *et al.* 2002; Martins *et al.* 2010; Martins 2012). The study of vascularization is an important tool to recognize the nature of structures and organs, which has been used for a long time and, at times, may identify evolutionary novelties (Aguiar-Dias *et al.* 2011; Gama *et al.* 2016).

The present study showed that the basal genera have vascular remnants in the peduncle of some interpetiolar colleters and that the vascularization was lost during Apocynaceae evolution. One possibility for the reduction and loss of the vascular system may be related to the extremely small size of the stipules and, consequently, the interpetiolar colleters. It is well known that the quantity of vascular tissue is proportional to the size of the structure (Carlquist 1969) and the procambium differentiation might be suppressed by a very reduced number of mesophyll cell layers (Puri 1951). Another possibility is that the formation of the secretory portion of the colleters suppresses the differentiation of the procambium since we observed the interruption of the vascularization of *Mussaenda* stipules and Apocynaceae bracteoles just below the secretory tip in both.

Our data demonstrate the homology between the interpetiolar colleters of Apocynaceae and the interpetiolar stipules found in Gentianales (Bremer and Struwe 1992), mainly in Rubiaceae (Robbrecht 1988), because they follow the same pattern, developing from protrusions of the petiole base that mostly merge into a stipular sheath. Even though the colleters of the petiole, leaf blade, and calyx are anatomically similar to stipular ones, their origin is distinct. In addition, vascular tissues were observed exclusively in the stipular colleters and the presence of vascularization was the reason that led Woodson and Moore (1938) to propose the stipular theory for colleters in Apocynaceae. Thus the stipular nature of the colleters in this family must be verified through developmental studies and this hypothesis should not be extended to the colleters occuring in other positions of the leaf nor to trichomatous colleters (in other orders; see Lacchia *et al.* 2016 and references therein), once trichomes are just epidermal projections (Fahn 1990), not representing projections of organs, as the stipules.

Further studies including a larger number of taxa from Apocynaceae and other Gentianales are needed to reevaluate the presence of stipules in the non-Rubiaceae families of the order and gene expression analyses may perhaps elucidate the factors responsible for the morphological evolution of the stipules in this group.

## Conclusions

In conclusion, most morphological and anatomical studies have described the family Apocynaceae as exstipulate with few representatives bearing stipules; however our study demonstrates that the condition may be reversed with the presence of stipules modified into interpetiolar colleters in most representatives of the family and their secondary loss may have occurred in a few genera. The same may have occurred in other families of the order, being necessary to analyze leaf ontogeny in these families to reevaluate the stipule evolution in Gentianales.

## Sources of Funding 

Our work was funded by FAPESP (proc. 02/11881-3, 04/09729-4, Biota/FAPESP proc. 03/12595-7) and Universidade de São Paulo.

## Contributions by the Authors

D.D. designed the research, B.A.R. and D.D. performed experiments and analyzed the data, and N.V.C. and D.D. interpreted the results and wrote the paper.

## Conflicts of Interest Statement

None declared.
